# The Expression of Tax and HBZ Genes in Serum-Derived Extracellular Vesicles From HTLV-1 Carriers Correlates to Proviral Load and Inflammatory Markers

**DOI:** 10.3389/fmicb.2022.881634

**Published:** 2022-05-02

**Authors:** Debora Glenda Lima de La-Roque, Elaine Vieira Santos, Evandra Strazza Rodrigues, Péricles Natan Mendes da Costa, Verônica Soares Brauer, Fausto Almeida, Tissiana Marques de Haes, Osvaldo Massaiti Takayanagui, Dimas Tadeu Covas, Simone Kashima

**Affiliations:** ^1^Regional Blood Center of Ribeirão Preto, University of São Paulo, São Paulo, Brazil; ^2^Medical School of Ribeirão Preto, University of São Paulo, São Paulo, Brazil

**Keywords:** HTLV-1, retrovirus, deltaretrovirus, neglected diseases, tax, HBZ

## Abstract

Human T-lymphotropic virus 1 (HTLV-1) is the etiologic agent of adult cell leukemia/lymphoma (ATL) and HTLV-1 associated myelopathy/tropical spastic paraparesis (HAM/TSP). One of the major questions in HTLV-1 studies is related to the understanding of causes that lead to different clinical manifestations. However, it is well known that the viral genes tax and HTLV-1 basic leucine zipper factor (HBZ) are related to viral infectivity and the development of neurological and hematological diseases. Currently, there is evidence that HTLV-1 infected cells can release small extracellular vesicles (sEVs) involved in the mechanisms of viral particles spreading. Therefore, we evaluated the expression levels of tax and HBZ viral transcripts in serum-derived sEVs from HTLV-1 carriers, as well as the role of these vesicles in the modulation of the immune response. Three HAM/TSP carriers presented detectable levels of tax and HBZ transcripts in sEVs and were positively correlated to the proviral load (PVL) in peripheral blood mononuclear cells (PBMCs). The viral transcripts were only detectable in individuals with a PVL higher than 6,000/10^5^ PBMCs. Additionally, it was observed that HBZ presented a 2–12-folds increase over tax expression units. Gene expression and secretory protein analysis indicated that PBMCs from blood donors and HTLV-1 carriers exposed to increasing doses of tax+ HBZ+ sEVs showed a dose-dependent increase in interferon (IFN)-γ and interleukin (IL)-8 transcripts and proteins. Interestingly, the increase in IL-8 levels was close to those seen in HTLV-1-infected PBMCs with high PVL. Taken together, these findings indicate that the expression of viral transcripts in serum-derived sEVs of HTLV-1 carriers is related to the PVL presented by the infected individual. Additionally, tax+ HBZ+ sEVs can induce the production of inflammatory cytokines in patients with low PVL, which may be related to the development of symptoms in HTLV-1 infection.

## Introduction

Human T-lymphotropic virus 1 (HTLV-1) was the first human retrovirus to be identified (Poiesz et al., 1980). It is mainly associated with two clinical diseases: the hematologic malignancy called adult T-cell leukemia/lymphoma (ATLL) and the neurodegenerative disease called HTLV-1-associated myelopathy/tropical spastic paraparesis (HAM/TSP) (Hinuma et al., 1981; Akizuki et al., 1987). Among HTLV-1 regulatory and accessory genes, tax and HTLV-1 basic leucine zipper factor (HBZ) stand out as potential markers for disease progression due to their association with viral infectivity and the expansion and survival of leukemic cells (Enose-Akahata et al., 2017). Tax is a highly immunogenic nuclear protein. In addition to promoting viral transcription in the 5' LTR region, it causes alterations in the cell cycle and in the classical nuclear factor kappa B (NFκB) pathway (Grassmann et al., 2005). In contrast, HBZ suppresses the transcription of viral genes with a promoter region in the 3′ LTR region and suppresses the classical NFκB pathway (Enose-Akahata et al., 2017).

Human T-lymphotropic virus 1 virions are spherical and pleomorphic in structure, with an average size of 100–120 nm. HTLV-1 virions share similar size and biogenesis to small extracellular vesicles (sEVs). EV is a term used to define particles secreted by prokaryotic and eukaryotic organisms, delimited by a lipid bilayer, and incapable of replicating (Théry et al., 2018). EV represents an important communication mechanism between cells, especially immune cells (Colombo et al., 2014). In retroviruses, EVs play roles in the pathogenesis, promoting the infection of new cells and the development of immune responses as they carry nucleic acids and viral proteins that trigger different processes in the recipient cells (Rezaie et al., 2021).

In recent years, several studies about HTLV-1 infection demonstrated alterations in the functions and cargo of EVs released by infected cells. Tax protein and viral transcripts of tax, env, and HBZ have been described in EVs derived from the cerebrospinal fluid of patients with HAM/TSP, as well as from the culture supernatant of infected cell lines (Jaworski et al., 2014; Anderson et al., 2018; Pinto et al., 2019). Unlike other viral infections, EVs released by HTLV-1-infected cells are not infectious. However, these particles are able to increase viral infectivity by promoting contact between infected and healthy cells (Pinto et al., 2019).

In functional evaluations of EVs from HTLV-1-infected cells, it was reported that tax-positive EVs from the cerebrospinal fluid (CSF) of patients with HAM/TSP and infected cell lineage culture supernatant are related to HTLV-1-specific immune response and contribute to characteristic chronic inflammation in patients with HAM/TSP (Anderson et al., 2018; Otaguiri et al., 2018). Another study revealed that EVs from infected cell lines can induce interleukin (IL)-8 cytokine expression, tissue damage, and viral spread in cells commonly associated with the neurovascular unit, such as astrocytes and monocytic cell-derived macrophages (Pinto et al., 2021). Furthermore, tax-positive EVs isolated from leukemic cells of patients with ATLL caused misregulation in mesenchymal stromal cells, favoring the establishment of leukemic cells (El-Saghir et al., 2016).

Despite growing evidence that HTLV-1 infected cells release EVs related to viral dissemination and pathogenesis mechanisms, there are few studies regarding plasma or serum EVs. In this context, the hypothesis that circulating EVs from HTLV-1 infected individuals act as delivery vehicles for molecules that contribute to the pathogenesis of HAM/TSP. This study evaluated the expression levels of the viral genes tax and HBZ in serum-derived EV of HTLV-1-infected individuals, as well as their immunomodulatory potential, measured by their ability to stimulate the release of inflammatory cytokines commonly altered in HAM/TSP individuals.

## Results

### Characterization of Participants According to Age and Proviral Load

**Figure 1:**
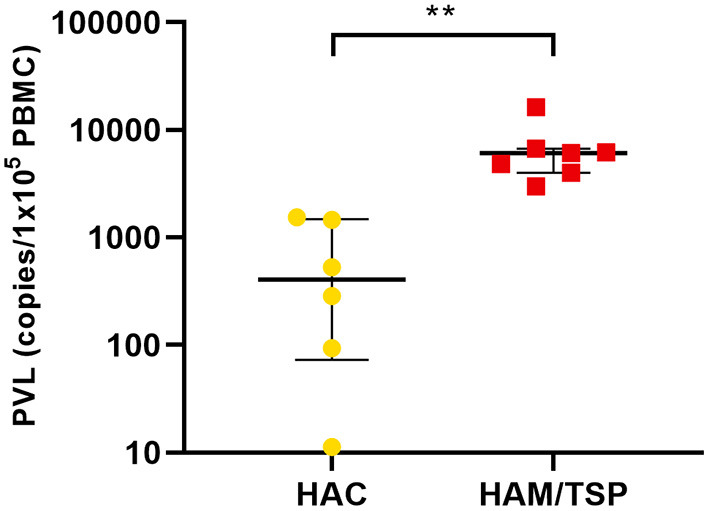
Human T-lymphotropic virus 1 (HTLV-1) proviral load (PVL) in patients with HTLV-1 asymptomatic carriers (HAC) and HTLV-1 associated myelopathy/tropical spastic paraparesis (HAM/TSP). The PVL is expressed by the median value of the estimated number of HTLV-1 provirus copies integrated in 10^5^ peripheral blood mononuclear cells (PBMCs) with interquartile range (IQR) for patients with HAC (*n* = 6) and HAM/TSP (*n* = 7). Statistical analysis was performed by non-parametric Mann–Whitney test: **p < 0.01.

In this study, 26 peripheral blood samples were collected (13 blood donors, 6 HTLV-1 asymptomatic carriers (HAC), and 7 HAM/TSP individuals). The distribution of the participants according to gender and age is shown in Supplementary Table 1. HAC and HAM/TSP individuals presented a mean age of 46 years (±17 years) and 56 years (±12 years) (Supplementary Table 1), respectively, with a mean proviral load (PVL) of 652.7 copies/10^5^ PBMCs and 6,727.5 copies/10^5^ PBMCs (Table 1), respectively. Figure 1 shows the distribution of PVL among infected groups. Patients with HAM/TSP presented higher PVL when compared with HAC (*p* = 0.0025, Mann–Whitney test). Those findings are also observed in other studies and reflect the state of infection in each patient (Olindo et al., 2005; Iwanaga et al., 2010; dos Furtado et al., 2012).

**Table 1:** Comparison between tax and HBZ messenger RNA (mRNA) expression in small extracellular vesicles (sEVs) and the proviral load (PVL) of human T-lymphotropic virus 1 (HTLV-1)-infected individuals.

	**mRNA tax**	**mRNA HBZ**	**PVL (HTLV-1 copies/10^**5**^ PBMC)**	**mRNA HBZ/mRNA tax**
CTRL 1	0	0	N/A	0
CTRL 2	0	0	N/A	0
CTRL 3	0	0	N/A	0
CTRL 4	0	0	N/A	0
CTRL 5	0	0	N/A	0
CTRL 6	0	0	N/A	0
CTRL 7	0	0	N/A	0
CTRL 8	0	0	N/A	0
CTRL 9	0	0	N/A	0
CTRL 10	0	0	N/A	0
CTRL 11	0	0	N/A	0
CTRL 12	0	0	N/A	0
CTRL 13	0	0	N/A	0
HAC 1	0	0	285.31	0
HAC 2	0	0	527.64	0
HAC 3	0	0	1,457.31	0
HAC 4	0	0	93.40	0
HAC 5	0	0	1,541.48	0
HAC 6	0	0	11.31	0
HAM/TSP 1	6,548.94	64,725.48	16,244.89	9.88
HAM/TSP 2	473.44	5,545.29	6,708.24	11.71
HAM/TSP 3	0	0	4,007.81	0
HAM/TSP 4	435.54	995.14	6,097.13	2.28
HAM/TSP 5	0	0	4,830.14	0
HAM/TSP 6	0	0	2,997.14	0
HAM/TSP 7	0	0	6,207.12	0

*N/A, Not applicable*.

### Characterization of Circulating EVs From Blood Donors and HTLV-1-Infected Individuals

**Figure 2:**
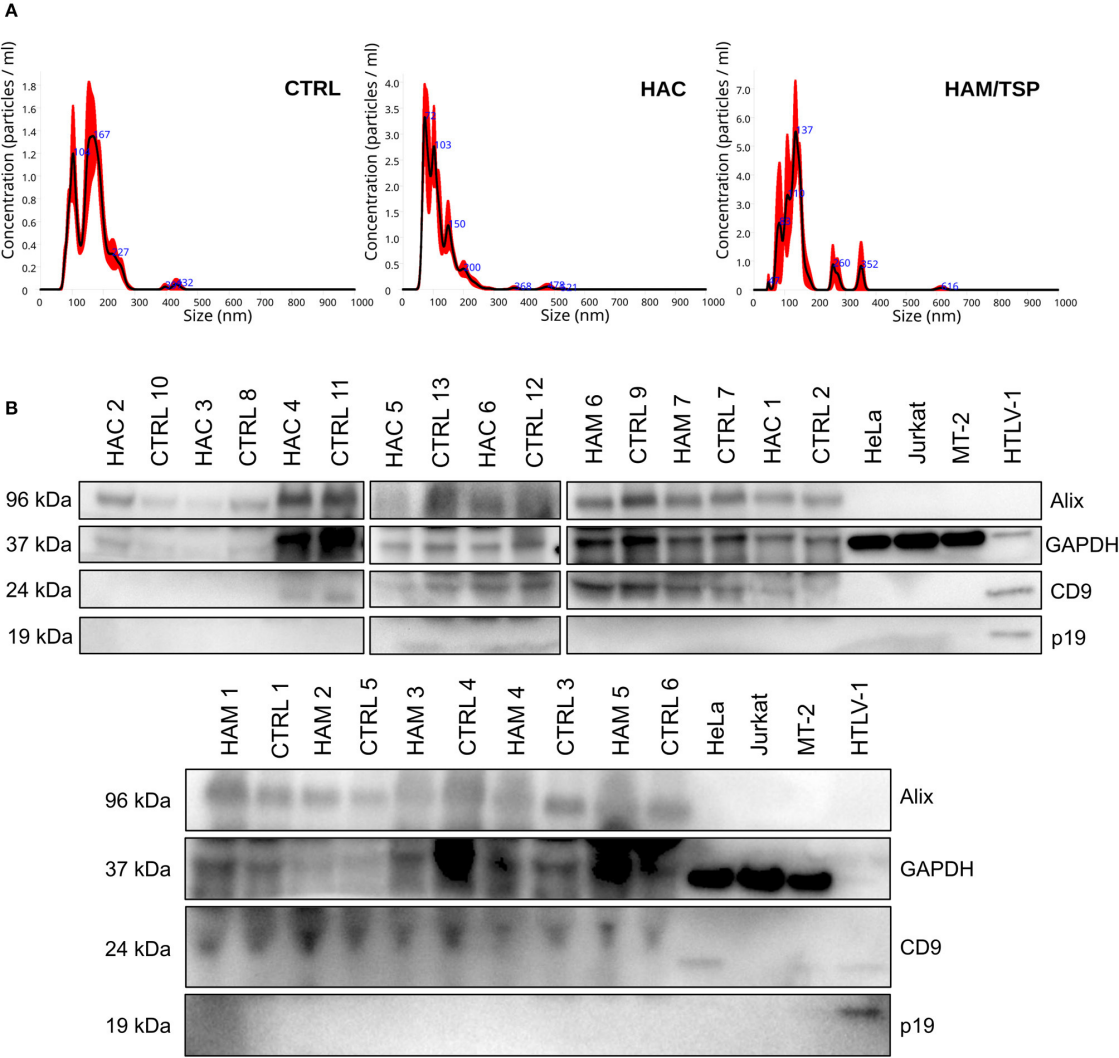
Serum-derived small extracellular vesicles (sEVs) characterization by size and protein markers. Graphic **(A)** demonstrates the size distribution of suspended particles from blood donors (CTRL), HAC and HAM/TSP individuals, respectively. **(B)** Western Blot analysis for Alix (96 kDa), CD9 (24 kDa), p19 (kDa), and GAPDH (37 kDa) in sEV suspensions from serum samples of HAM/TSP (HAM1–HAM7), HAC (HAC1–HAC6), and control (CTRL1–CTRL13) groups. The protein extract from HTLV-1 virions was used as positive control for p19.

Previous data from our group showed that HTLV-1-infected cell lines release EVs with viral cargo and can induce the production of proinflammatory cytokines (Otaguiri et al., 2018). To verify if this is also valid for circulating EVs from HTLV-1 carriers, sEVs were isolated from the serum of HAC, HAM/STP individuals, and blood donors using the Exoquick exosome precipitation solution (System Biosciences). Serum-derived sEVs from all three groups presented a modal diameter up to 166.2 nm when evaluated by nanoparticle tracking analysis (Figure 2A). In addition, western blot analysis detected classic EV markers (Alix and CD9) and the absence of the viral protein p19 (Figure 2B). Since sEVs and HTLV-1 viral particles share the endosomal pathway during biogenesis, the exclusion of p19 in sEV extracts indicates none or low levels of contamination with HTLV-1 virions. The presence of EV markers in the protein extract of HTLV-1 particles portrays the shared pathway (Figure 2B).

### Tax and HBZ Messenger RNA Detection in Serum-Derived sEVs

**Figure 3:**
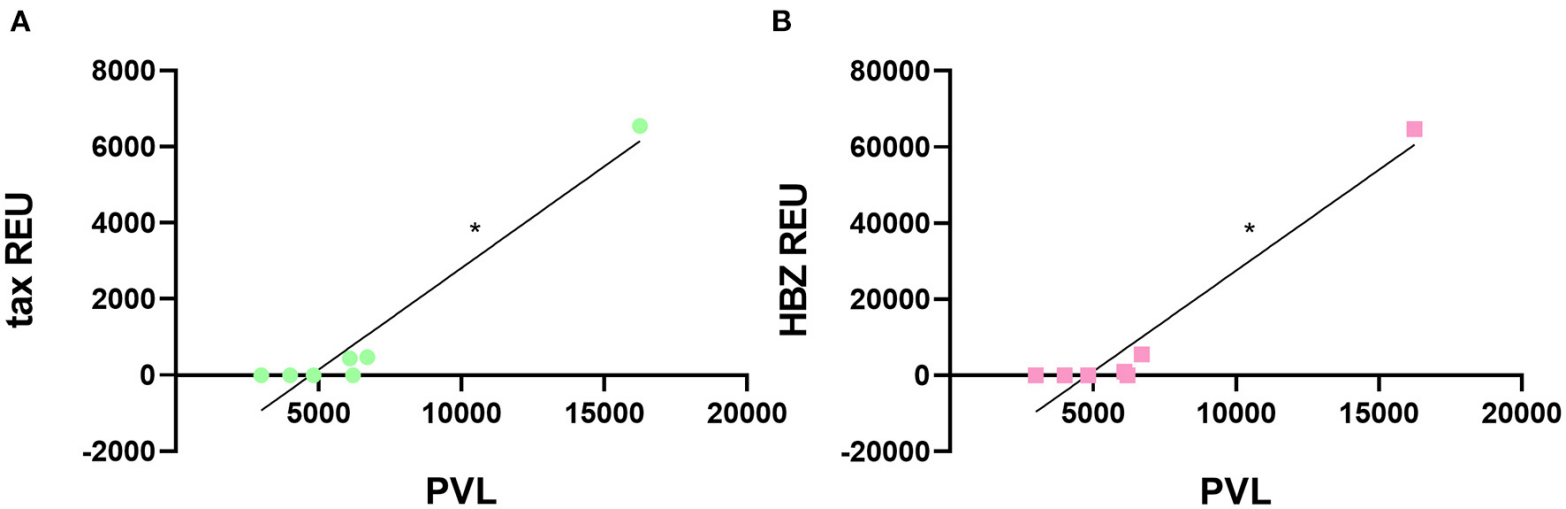
Correlation between HTLV-1 PVL and viral gene expression in sEVs. The HTLV-1 PVL was positively correlated to **(A)** tax and **(B)** HBZ transcripts in sEVs. *p < 0.05.

Following sEVs characterization, we sought to determine the expression levels of tax and HBZ viral messenger RNA (mRNA) in serum-derived sEVs from HTLV-1 carriers. A quantitative real-time PCR (RT-qPCR) analysis was performed, and the amplification data were normalized to the expression of the β-actin gene within sEVs. From all 13 serum samples of HTLV-1 carriers, only three presented sEVs with detectable levels of tax and HBZ mRNA (Table 1). These samples corresponded to HAM/TSP individuals with PVL higher than 6,000 HTLV-1 copies/10^5^ PBMCs. Interestingly, HAM/TSP 1 showed PVL three times higher than the other patients with HAM/TSP at blood sampling. The notable PVL was accompanied by higher tax and HBZ expressions in sEVs when compared with other individuals—especially HBZ. In all three samples, the HBZ mRNA/tax mRNA ratio demonstrated 2.2 to 11.7 fold increase in HBZ expression over tax expression (Table 1). Therefore, the correlation between the sEVs viral transcripts and the HTLV-1 PVL was measured. As expected, our results indicated a positive correlation between tax and HBZ mRNA in sEVs and the patient's PVL (Spearman's *r* = 0.0381) (Figure 3).

### sEVs Immunomodulation Assay

Finally, we investigated whether or not serum-derived tax+ HBZ+ sEV triggers the transcription and secretion of inflammatory cytokines in PBMCs. Increasing doses of control sEV and tax+ HBZ+ sEV (500, 1,000, and 2,000 μg/ml) from HAM/TSP1 were co-cultivated with heterologous naive PBMCs and heterologous HTLV-1-infected PBMCs. Simultaneously, sEVs from CTRL1 were co-cultivated under the same conditions as a negative control for immunomodulation. The production of inflammatory cytokines (interferon (IFN)-γ, tumor necrosis factor (TNF)-α, IL-4, IL-6, IL-8, IL-10, and C–X–C motif chemokine 10 [CXCL10/IP-10]) was evaluated by quantitative PCR (qPCR) and the Luminex xMAP assay. The selected cytokines correspond to those commonly altered in symptomatic HTLV-1 carriers (Futsch et al., 2018).

**Figure 4:**
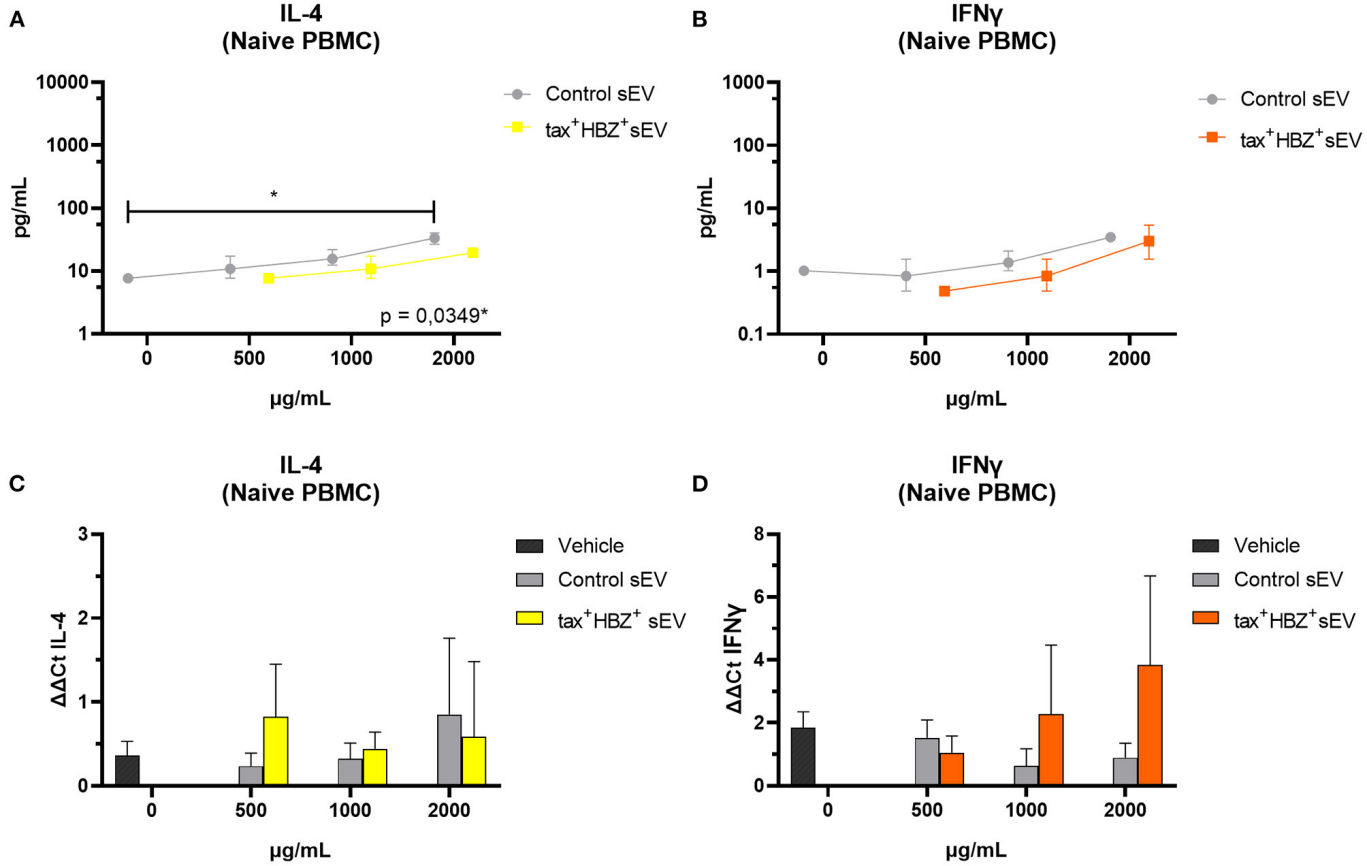
Inflammatory cytokines expression and secretion in naive PBMCs treated with tax+ HBZ+ sEV. The secretion of interleukin (IL)-4 and interferon (IFN)-γ **(A,B)** was analyzed in total protein extract of naive PBMCs (*n* = 3) treated with 500, 1,000, and 2,000 μg/ml of tax+ HBZ+ sEV. Graphs A and B show the average IL-4 and IFN-γ concentration ± standard deviation (SD) in pg/ml in response to each sEV treatment. The same conditions were analyzed by quantitative PCR (qPCR). Graphs **(C,D)** show the average IL-4 and IFN-γ mRNA relative expression (ΔΔCt) ± SD in response to each sEV treatment. Statistical analysis was performed by Kruskal–Wallis with Dunn's *post-hoc* test: *p < 0.05.

Regarding naive PBMCs, it was observed that those treated with 2,000 μg/ml of control sEV presented higher IL-4 protein levels than untreated PBMCs (Kruskal–Wallis *post-hoc* Dunn's test, *p* = 0.0349) (Figure 4A). However, IL-4 transcripts levels did not alter significantly under the same treatment (Figure 4C). Tax+ HBZ+ sEV did not alter significantly any cytokine levels. Moreover, IFN-γ transcripts and protein levels increased in a dose-dependent manner after PBMCs treatment with tax+ HBZ+ sEV (Figures 4B,D).

As for the assays with infected cells, two PBMC samples were used: HAC 6, which corresponded to a patient with low PVL (11.3 HTLV-1 copies/10^5^ PBMCs), and HAC 7, a patient with high PVL (5,806.3 copies/10^5^ PBMCs). Those samples were used to evaluate the production of inflammatory cytokines by the adaptive immune response to HTLV-1.

**Figure 5:**
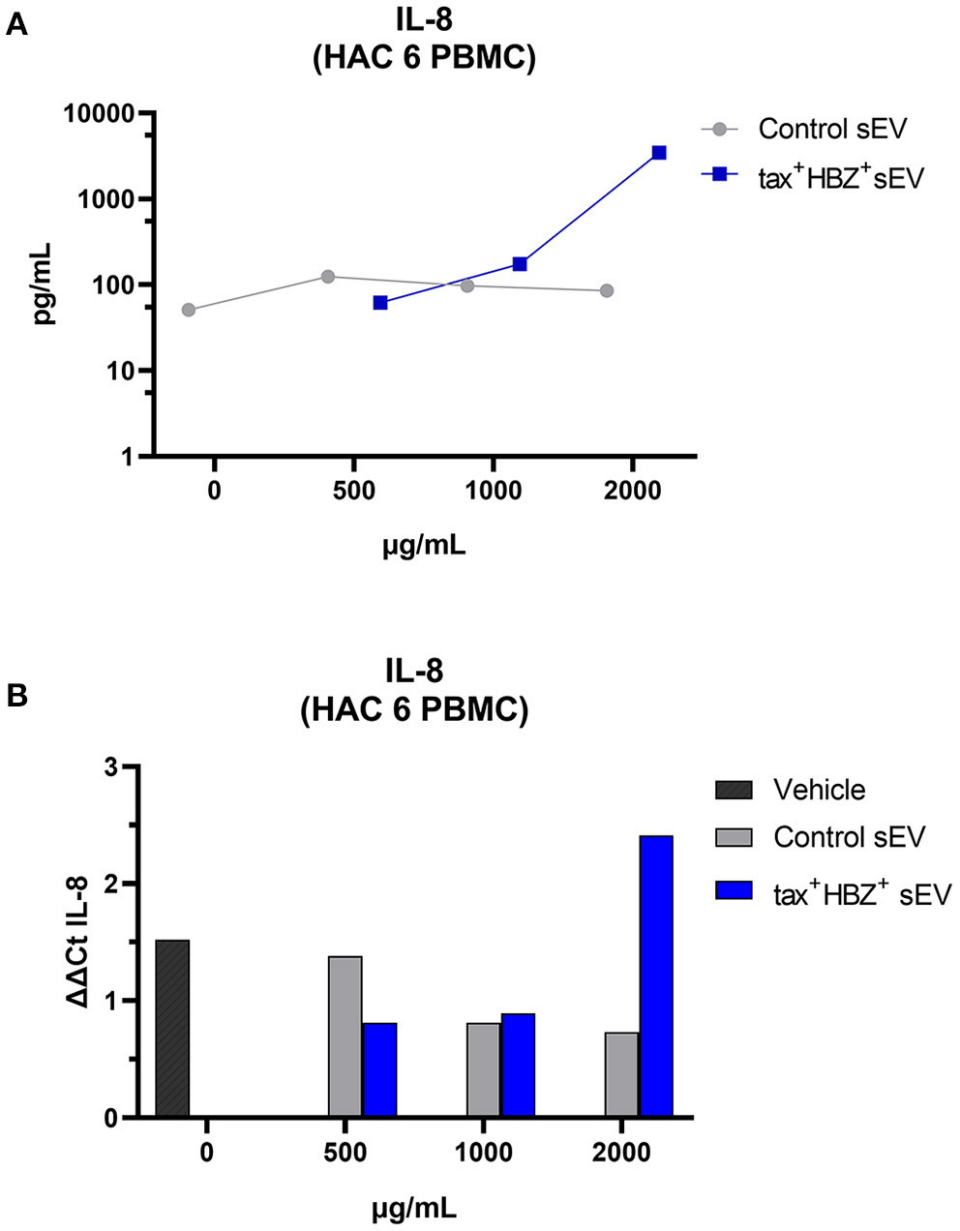
Interleukin-8 expression and secretion in HTLV-1-infected PBMCs treated with tax+ HBZ+ sEV. **(A)** The secretion of IL-8 was analyzed in the total protein extract of HAM/TSP PBMCs (*n* = 1) treated with 500, 1,000, and 2,000 μg/ml of tax+ HBZ+ sEV. Graph A shows the average IL-8 concentration in pg/ml in response to each sEV treatment. **(B)** The same conditions were analyzed by qPCR. Graph B shows the average IL-8 relative expression (ΔΔCt) in response to sEV treatment.

No dose of control sEV altered the transcription or secretion of the analyzed cytokines in both infected PBMCs. When compared with naive PBMCs, HAC 7 PBMCs showed much higher secretion of all inflammatory cytokines regardless of tax+ HBZ+ sEV treatment. However, the effects of tax+ HBZ+ sEV treatments on HAC 6 PBMCs followed a dose-dependent increase in IL-8 transcripts and protein levels when compared with the untreated PBMCs (Figures 5A,B). Interestingly, after treatment with 2,000 μg/ml of tax+ HBZ+ sEV, IL-8 levels were similar to those observed in PBMCs with high PVL (HAC 7) (Supplementary Figure 1). These data suggest an sEV-mediated increase in inflammatory responses.

## Discussion

The present work considered three aspects regarding serum-derived sEVs in HTLV-1 carriers: the presence of tax and HBZ transcripts; the correlation between tax and HBZ transcripts; patient's PVL and sEV tax+ HBZ+ immunomodulatory potential. We believe that the functional study of EVs in HTLV-1 infection can contribute to the understanding of the pathophysiology of associated diseases, such as HAM/TSP.

In the first place, we evaluated HTLV-1 PVL in PBMCs of symptomatic and asymptomatic individuals, since PVL is a parameter used to monitor HTLV-1-disease progression (Olindo et al., 2005). As expected, patients with HAM/TSP presented higher PVL in comparison with HAC. This finding is also observed in other HTLV-1 studies and demonstrates the advanced state of infection in symptomatic patients (Nagai et al., 1998; Orland et al., 2003; Olindo et al., 2005; Iwanaga et al., 2010; dos Furtado et al., 2012).

Next, we explored the possibility of a high PVL interfering in serum-derived EVs content. The outcomes showed that tax and HBZ mRNA were only detectable in individuals with PVL above 6,000 copies/10^5^ PBMC, and were positively correlated with PVL. Additionally, HBZ transcripts were about 2–11 times more expressed than tax transcripts. The detection of viral material in sEVs released by HTLV-1 infected cells has been described previously (Jaworski et al., 2014; Anderson et al., 2018; Otaguiri et al., 2018; Pinto et al., 2019). Additionally, the higher expression of HBZ compared with tax corroborates other studies that evaluated tax and HBZ load in PBMC (Saito et al., 2009). The detection of the tax and HBZ genes in PBMC is largely associated with the development of symptoms. These findings suggest that HTLV-1-infected cells may be using the EVs communication pathway to spread viral content to uninfected cells, possibly aiding in the perpetuation of the chronic inflammatory condition seen in patients with HAM/TSP.

To test this hypothesis, we investigated the immunomodulatory potential of serum-derived tax+ and HBZ+, measured by its ability to stimulate the release of inflammatory cytokines in PBMCs from blood donors (naive PBMCs) and patients with HAC (HTLV-1+ PBMCs) with different PVL. In naive PBMCs, the data suggest an increase in protein levels and transcription of IFN-γ after co-cultivation with EV tax+ HBZ+, though this is not statistical significant. Recent studies have shown that EVs are able to induce a pathogen-specific response in immune cells in studies regarding *Salmonella typhimurium* (Hui et al., 2021), human immunodeficiency virus (Sampey et al., 2016), hepatitis B virus (Kouwaki et al., 2016), and Epstein-Barr virus (Mrad et al., 2021).

In HTLV-1 infection, Jaworski et al. (2014) demonstrated that the co-culture of dendritic cells with EV tax+ causes an increase in the secretion of inflammatory cytokines common to the Th1 response. In lymphocytes, Anderson et al. (2018) and Otaguiri et al. (2018) demonstrated that these EVs cause an increase in the secretion of cytokines IFN-γ and TNF-α and sensitization of cytotoxic T lymphocytes to specific responses to Tax. Regarding our data, it is important to emphasize the difference between sources of EVs in this study and others that identified the increase in IFN-γ with a similar co-culture. While Jaworski et al. (2014), Anderson et al. (2018), and Otaguiri et al. (2018) obtained EVs from the supernatant of cultured cells infected with HTLV-1, our study obtained EVs from peripheral blood, which carries EVs from different cells. Our data suggest that the use of biological fluids as a source of EV provides results closer to the *in vivo* immunomodulatory properties of EV.

What is more, in naive PBMCs, only IL-4 showed a significant increase post-treatment with 2,000 μg/ml of control sEV. In naive PBMCs, IL-4 release is associated with mastocytes, basophils, and eosinophils in response to helminths and allergens, resulting in Th2 response polarization (Abbas and Lichtman, 2005). Conflicting with our results, Gehrmann et al. (2011) reported that EVs from the plasma of healthy individuals did not alter IL-4 expression in PBMCs. We elaborate on two hypotheses for these results. In the first one, there was a delivery of mRNA by sEVs of this particular blood donor to the PBMCs used in the assay, resulting in a detectable increase by qPCR. Our second hypothesis is that the sEVs from this donor presented an unknown antigen that causes the increase of IL-4 in *naive* PBMCs. However, none of these hypotheses were explored in this article.

Regarding PBMCs from HAC, cells were classified into two groups: high (5,806.3 copies/10^5^ PBMCs) and low (11.3 copies/10^5^ PBMCs) PVL. In PBMCs with high PVL, an upregulation was observed in all analyzed cytokines, independent of sEV co-cultivation. These results were expected since it is known that this group of cytokines is altered by chronic inflammation in symptomatic patients with high PVL (Futsch et al., 2018; Kagdi et al., 2018).

Notably, PBMC with low PVL presented a dose-dependent increase of both IL-8 transcripts and proteins after cocultivation with sEV tax+ HBZ+. The IL-8 protein is a chemotactic agent, together with other chemokines, it controls the trafficking of immune cells, such as neutrophils and T lymphocytes during inflammation (Palomino and Marti, 2015). In HTLV-1 infection, IL-8 is commonly altered in patients with HAM/TSP (Futsch et al., 2018). This chemokine was previously associated with the presence of Tax protein in immortalized lymphocytes (Zhang and Sharma, 2000) but can also be suppressed through the HBZ suppression of the classical NFkB pathway (Zhao et al., 2009).

It is well known that EVs can transpose the blood-brain barrier carrying molecules, such as proteins and RNAs (Alvarez-Erviti et al., 2011). The EVs-mediated transport of altered proteins to the central nervous system is one of the mechanisms for neurodegeneration observed in diseases, such as Alzheimer's, Parkinson's, and Huntington's disease (Kalani et al., 2014). Concerning retrovirus infections, Hu et al. (2012) described that astrocyte-derived EVs cause neurotoxic effects in patients with HIV using opioids. Furthermore, Pinto et al. (2021) demonstrated an increase in IL-8 secretion after treatment of central nervous system cells with EVs released by an HTLV-1-infected cell line.

Our outcomes corroborate previous findings and support the idea that HTLV-1-infected cells use EVs-mediated communication to perpetuate chronic inflammation and may be involved in the development of neuroinflammation observed in HAM/TSP. This is the first work to describe the presence of viral genes in serum-derived sEVs of patients with HTLV-1. In summary, our data show that serum-derived sEVs can incorporate viral components during their biogenesis. This process is directly associated with the patient's PVL. The incorporation of viral components by EVs potentializes inflammation as it causes dysregulation of inflammatory cytokines in naive and activated PBMCs.

## Methods

### Sample Acquisition

Peripheral blood samples were collected from blood donors and HTLV-1-infected adult subjects of both sexes. Samples were fractionated into serum and PBMCs. Infected individuals were classified as HTLV-1 asymptomatic carriers (HAC) or HAM/TSP carriers according to the World Health Organization (WHO) diagnostic criteria.

### Cell Culture

In this work, cell lineages HeLa (ATCC-CCL-2), Jurkat (ATCC TIB-152), MT-2 (ECACC 08081401), and primary culture of human PBMCs were used. Cell culture media for HeLa expansion was D-MEM (Gibco) supplemented with 1% Penicillin-Streptomycin at 10,000 U/ml (Gibco) and 10% fetal bovine serum (Gibco). Jurkat, MT-2, and human PBMCs were cultured with RPMI 1640 supplemented with 1% Penicillin-Streptomycin at 10,000 U/ml (Gibco) and 10% fetal bovine serum (Gibco). All cells were cultivated at 37°C in a 95% humid atmosphere with 5% CO_2_.

### DNA HTLV-1 PVL

Human T-lymphotropic virus 1 PVL was quantified by multiplex qPCR as previously described (Rodrigues et al., 2022). Briefly, PBMCs were isolated by the density gradient method, using Ficoll Hypaque PLUS reagent (GE Healthcare) and genomic DNA extracted by the Gentra Puregene Blood kit (Qiagen). Using primers and probes to pol viral gene and the human β-globin endogenous gene, the number of integrated HTLV-1 provirus copies was determined using linear interpolation in a plasmidial standard curve. The PVL was calculated by the formula:


$$\begin{array}{l} PVL\;=\left(\frac{number\;of\;pol\;copies}{number\;of\;\beta -globin\;copies}\right)\times 2\times 10^{5} \end{array}$$

### EVs Isolation

Serum samples were preprocessed through centrifugation at 3,000 × g for 15 min to remove larger vesicles (apoptotic bodies). Then, exoquick reagent (System Biosciences) was used to enrich EVs from 1.5 ml of human serum according to the manufacturer's recommendations. Briefly, serum samples were incubated with the one-fourth volume of the Exoquick polymer at 4°C for 1 h. The samples were then centrifuged at 1,500 × g for 30 min and the resulting pellet was resuspended in 400 μl of PBS for the following analyses.

### EVs Characterization

The isolated EVs were characterized by size and protein markers. EVs concentration and size distribution were accessed by nanoparticle tracking analysis (NTA) (NS300 equipment, NanoSight). Images were acquired for 15 s in triplicate with the following parameters: scientific Complementary Metal-Oxide-Semiconductor (sCMOS) camera, green laser (532 nm), camera shutter 1,300, camera gain 512, and detection threshold 8. Protein markers were accessed by western blot. For protein extraction, 50 μl of EVs suspension were disrupted by 100 μl of radioimmunoprecipitation assay (RIPA) buffer (Sigma-Aldrich) plus 1% protease inhibitors (Sigma-Aldrich). Protein concentration was determined by the Bradford microplate assay (Bio-rad), and 30 μg of total proteins were applied in 4–20% Mini-PROTEAN® TGX™ Precast Protein Gels (Bio-rad). The migrated proteins were transferred to a polyvinylidene fluoride (PVDF) membrane (GE Healthcare) previously activated in methanol (Merck), followed by blocking with 10% milk (w/v) (Bio-rad) and 0.1% Tween 20 (v/v) (Sigma-Aldrich) in Tris Buffered Saline solution (TBS, 50 mM Tris, 150 mM NaCl). The primary antibodies used were 1:1,000 anti-Alix (Cell Signaling), 1:1,000 anti-GAPDH (Cell Signaling), 1:1,000 anti-CD9 (Abcam), and 1:1,000 anti-HTLV-1 p19 (Abcam).

### Viral Gene Expression in EVs

Extracellular vesicle RNA extraction was performed by lysis and phase separation followed by purification using the QIAamp Viral RNA Mini Kit (QIAGEN). Precipitated EV was added with 750 μl of TRIzol LS reagent (Thermofisher Scientific) and 150 μl of chloroform. The samples were vigorously vortexed and incubated at room temperature for 10 min. Phase separation was observed after centrifugation at 12,000 × g for 15 min at 4°C. For each 200 μl of aqueous phase obtained, 1 μl of linear polyacrylamide was added to enrich the RNA yield (GenElute LPA, Sigma-Aldrich) and 200 μl of isopropanol (Merck). After overnight incubation, samples were loaded onto the column provided by the QIAamp Viral RNA Mini kit, and extraction proceeded according to the manufacturer's instructions.

Complementary DNA (cDNA) synthesis was performed from 200 ng of RNA using the High-Capacity cDNA Reverse Transcription kit (Thermofisher Scientific) and specific antisense primers for tax (5′-GAGAAACTTACCCATGGTGTTGG-3′ and nucleotides 5,169–5,191), HBZ (5′-GAGCCGATAACGCGTCCAT-3′ and nucleotides 1,086–1,104), and the endogenous β-actin genes (5′-CCAGGGCAGTGATCTCCTTCT-3′ and nucleotides 1,025–1,045).

The relative quantification of viral genes was performed by singleplex qPCR, using specific primers and probes for tax (5′-ATCCCGTGGAGACTCCTCAA-3′, sense, nucleotides 5,098–5,117, 5′-GAGAAACTTACCCATGGTGTTGG-3′, antisense, nucleotides 5,169–5,191, and 5′-FAMAGCTGCATGCCCAAGAMGB-3′, probe, nucleotides 5,115–5,130), HBZ (5′-TACATCGTCACGCCCTACTGG-3′, sense, nucleotides 1,026–1,046, 5′-GAGCCGATAACGCGTCCAT-3′, antisense, nucleotides 1,086–1,104, and 5′-FAMATCAGATCACCTGGGACCMGB-3′, probe, nucleotides 1,062–1,079), and endogenous β-actin genes. The amplification of the endogenous gene used the Human ACTB Endogenous Control (Applied Biosystems). The reactions were performed in duplicate using TaqMan Universal PCR Master Mix (Applied Biosystems) and the ABI 7500 sequence detection platform (Applied Biosystems). The results of gene expression of tax and HBZ were expressed in Relative Expression Units (REUs), according to the calculation described (Albesiano et al., 2003):


$$\begin{array}{l} \Delta Ct=\left(Ct\;of\;the\;target\;gene\right)\\ \;-\left(geometric\;mean\;of\;the\;Cts\;of\;the\;reference\;gene\right)\\ REU=\frac{10,000}{2^{\Delta Ct}} \end{array}$$

### Functional Assays

Peripheral blood mononuclear cells were isolated by the density gradient method, using Ficoll Hypaque PLUS reagent (GE Healthcare). A total of 10^5^ cells were plated in 24-well plates (Greiner) and co-cultured with increasing doses of sEVs (500, 1,000, and 2,000 μg/ml) isolated from a patient with HAM/TSP (HAM/TSP 1) and a blood donor (CTRL 1), both paired by sex and age. In addition, we co-cultured PBMCs with the Exoquick polymer used to precipitate sEV (vehicle) as a negative control. For the assay, RPMI 1640 medium (Gibco) was used supplemented with 1% Penicillin-Streptomycin at 10,000 U/ml (Gibco) and 10% exosome-depleted fetal bovine serum (Gibco). After 48 h at 37°C, cells and culture supernatant were collected and stored at −80°C.

Seven inflammatory cytokines and chemokines were evaluated in culture supernatant by the Luminex xMAP assay: INF-γ, TNF-α, IL-4, IL-6, IL-8, IL-10, and CXCL10/IP-10. Data acquisition was performed using the Luminex MAGPIX System-EMD Millipore equipment, and the acquired data were analyzed using the Milliplex Analyst v3.5 software (Millipore). The detection procedure was performed according to the manufacturer's instructions.

The altered analytes were confirmed by gene expression. PBMCs were submitted to RNA extraction by the RNeasy Mini Kit (Qiagen) according to the manufacturer's recommendations. cDNA was synthesized by reverse transcriptase reaction from 50 ng of RNA, using the High-Capacity cDNA Reverse Transcription kit (Thermofisher Scientific) according to the manufacturer's instructions. Gene expression analysis was performed using the TaqMan system, with specific probes for the INF-γ (Hs00989291_m1), IL-4 (Hs00174122_m1), and IL-8 (Hs00174103_m1) genes. The normalization of the reaction was performed by the endogenous β-actin gene (ACTB, Hs99999903_m1), and cytokine expression levels were calculated by the delta-delta Ct method.

### Statistical Analysis

For the confection of graphs and inferential analysis, we used the GraphPad Prism software, version 8.0.2. The difference between the PVL presented by symptomatic and asymptomatic participants was investigated by the Mann–Whitney test. The correlation between PVL and levels of viral transcripts in EV was evaluated by Spearman's correlation test. Finally, the response to EV treatment observed in PBMCs was assessed by the Kruskal–Wallis test. For all analyses, data were expressed as mean ± standard deviation (SD) and a bilateral value of *p* ≤ 0.05 was considered statistically significant.

## Data Availability Statement

The raw data supporting the conclusions of this article will be made available by the authors, without undue reservation.

## Ethics Statement

The studies involving human participants were reviewed and approved by Regional Ethics Committee at the General Clinical Hospital of the School of Medicine of Ribeirão Preto, University of São Paulo (Process Number: 9179/2019). The patients/participants provided their written informed consent to participate in this study.

## Author Contributions

DL-R idealized the proposal, performed major experiments and analysis and wrote the original draft. ES idealized the proposal, performed major experiments, and reviewed the original draft. ER established and performed the proviral load measurement. PC contributed to western blotting experiments and immunomodulatory assays, and wrote the original draft. VB and FA performed all the nanoparticle tracking analysis. TH and OT realized the diagnosis of HAM/TSP and ambulatory follow up of HTLV-1 infected individuals. DC idealized the proposal, promoted the funding acquisition and guaranteed the laboratory structure for research execution. SK idealized the proposal, supervised and reviewed the original draft. All authors contributed to manuscript revision, read, and approved the submitted version.

## Funding

This work was supported by Fundação de Amparo à Pesquisa do Estado de São Paulo (FAPESP 2013/08135-2, 2014/50947-7), Conselho Nacional de Desenvolvimento Científico e Tecnológico (CNPq number 465539/2014-9), Coordenação de Aperfeiçoamento de Pessoal de Nível Superior (CAPES 88887.320793/2019-00), and Fundação Hemocentro de Ribeirão Preto.

## Conflict of Interest

The authors declare that the research was conducted in the absence of any commercial or financial relationships that could be construed as a potential conflict of interest.

## Publisher's Note

All claims expressed in this article are solely those of the authors and do not necessarily represent those of their affiliated organizations, or those of the publisher, the editors and the reviewers. Any product that may be evaluated in this article, or claim that may be made by its manufacturer, is not guaranteed or endorsed by the publisher.

## References

[B1] Abbas A. K. Lichtman A. H. (2005). Imunologia Celular e Molecular. Rio de Janeiro: Elsevier. p. 576.

[B2] Akizuki S. Nakazato O. Higuchi Y. Tanabe K. Setoguchi M. Yoshida S.. (1987). Necropsy findings in HTLV-I associated myelopathy. Lancet 329, 156–157. 10.1016/S0140-6736(87)91984-2 2879986

[B3] Albesiano E. Messmer B. T. Damle R. N. Allen S. L. Rai K. R. Chiorazzi N. (2003). Activation-induced cytidine deaminase in chronic lymphocytic leukemia B cells: expression as multiple forms in a dynamic, variably sized fraction of the clone. Blood 102, 3333–3339. 10.1182/blood-2003-05-1585 12855567

[B4] Alvarez-Erviti L. Seow Y. Yin H. Betts C. Lakhal S. Wood M. J. A. (2011). Delivery of siRNA to the mouse brain by systemic injection of targeted exosomes. Nat. Biotechnol. 29, 341–345. 10.1038/nbt.1807 21423189

[B5] Anderson M. R. Pleet M. L. Enose-Akahata Y. Erickson J. Monaco M. C. Akpamagbo Y.. (2018). Viral antigens detectable in CSF exosomes from patients with retrovirus associated neurologic disease: functional role of exosomes. Clin. Transl. Med. 7, 24. 10.1186/s40169-018-0204-7 30146667 PMC6110307

[B6] Colombo M. Raposo G. Théry C. (2014). Biogenesis, secretion, and intercellular interactions of exosomes and other extracellular vesicles. Annu. Rev. Cell Dev. Biol. 30, 255–289. 10.1146/annurev-cellbio-101512-122326 25288114

[B7] dos Furtado M. S. B. S. Andrade R. G. Romanelli L. C. F. Ribeiro M. A. Ribas J. G. Torres E. B.. (2012). Monitoring the HTLV-1 proviral load in the peripheral blood of asymptomatic carriers and patients with HTLV-associated myelopathy/tropical spastic paraparesis from a Brazilian cohort: ROC curve analysis to establish the threshold for risk disease. J. Med. Virol. 84, 664–671. 10.1002/jmv.23227 22337307

[B8] El-Saghir J. Nassar F. Tawil N. El-Sabban M. (2016). ATL-derived exosomes modulate mesenchymal stem cells: potential role in leukemia progression. Retrovirology 13, 1–13. 10.1186/s12977-016-0307-4 27760548 PMC5070229

[B9] Enose-Akahata Y. Vellucci A. Jacobson S. (2017). Role of HTLV-1 Tax and HBZ in the pathogenesis of HAM/TSP. Front. Microbiol. 8, 1–10. 10.3389/fmicb.2017.02563 29312243 PMC5742587

[B10] Futsch N. Prates G. Mahieux R. Casseb J. Dutartre H. (2018). Cytokine networks dysregulation during HTLV-1 infection and associated diseases. Viruses 10, 691. 10.3390/v10120691 30563084 PMC6315340

[B11] Gehrmann U. Qazi K. R. Johansson C. Hultenby K. Karlsson M. Lundeberg L.. (2011). Nanovesicles from malassezia sympodialis and host exosomes induce cytokine responses–novel mechanisms for host-microbe interactions in atopic eczema. PLoS One 6:e21480. 10.1371/journal.pone.0021480 21799736 PMC3142114

[B12] Grassmann R. Aboud M. Jeang K.-T. (2005). Molecular mechanisms of cellular transformation by HTLV-1 Tax. Oncogene 24, 5976–5985. 10.1038/sj.onc.1208978 16155604

[B13] Hinuma Y. Nagata K. Hanaoka M. Nakait M. Matsumotot T. Kinoshita K.-I.. (1981). Adult T-cell leukemia: antigen in an ATL cell line and detection of antibodies to the antigen in human sera (immunofluorescence/lymphoid cell line/specific antibody/type C virus). Med. Sci. 78, 6476–6480. 10.1073/pnas.78.10.6476 7031654 PMC349062

[B14] Hu G. Yao H. Chaudhuri A. D. Duan M. Yelamanchili S. V. Wen H.. (2012). Exosome-mediated shuttling of microRNA-29 regulates HIV Tat and morphine-mediated neuronal dysfunction. Cell Death Dis. 3:e381–e381. 10.1038/cddis.2012.114 22932723 PMC3434655

[B15] Hui W. W. Emerson L. E. Clapp B. Sheppe A. E. Sharma J. del Castillo J.. (2021). Antigen-encapsulating host extracellular vesicles derived from Salmonella-infected cells stimulate pathogen-specific Th1-type responses *in vivo*. PLoS Pathog. 17, e1009465. 10.1371/journal.ppat.1009465 33956909 PMC8101724

[B16] Iwanaga M. Watanabe T. Utsunomiya A. Okayama A. Uchimaru K. Koh K.-R.. (2010). Human T-cell leukemia virus type I (HTLV-1) proviral load and disease progression in asymptomatic HTLV-1 carriers: a nationwide prospective study in Japan. Blood 116, 1211–1219. 10.1182/blood-2009-12-257410 20448111

[B17] Jaworski E. Narayanan A. Van Duyne R. Shabbeer-Meyering S. Iordanskiy S. Saifuddin M.. (2014). Human T-lymphotropic virus type 1-infected cells secrete exosomes that contain tax protein. J. Biol. Chem. 289, 22284–22305. 10.1074/jbc.M114.549659 24939845 PMC4139239

[B18] Kagdi H. Demontis M. A. Ramos J. C. Taylor G. P. (2018). Switching and loss of cellular cytokine producing capacity characterize in vivo viral infection and malignant transformation in human T- lymphotropic virus type 1 infection. PLoS Pathog. 14, e1006861. 10.1371/journal.ppat.1006861 29444188 PMC5828519

[B19] Kalani A. Tyagi A. Tyagi N. (2014). Exosomes: mediators of neurodegeneration, neuroprotection and therapeutics. Mol. Neurobiol. 49, 590–600. 10.1007/s12035-013-8544-1 23999871 PMC3951279

[B20] Kouwaki T. Fukushima Y. Daito T. Sanada T. Yamamoto N. Mifsud E. J.. (2016). Extracellular vesicles including exosomes regulate innate immune responses to hepatitis B virus infection. Front. Immunol. 7, 335. 10.3389/fimmu.2016.00335 27630638 PMC5005343

[B21] Mrad M. F. Saba E. S. Nakib L. Khoury S. J. (2021). Exosomes from subjects with multiple sclerosis express EBV-derived proteins and activate monocyte-derived macrophages. Neurol. Neuroimmunol. Neuroinflam. 8, e1004. 10.1212/NXI.0000000000001004 34006621 PMC8130999

[B22] Nagai M. Usuku K. Matsumoto W. Kodama D. Takenouchi N. Moritoyo T.. (1998). Analysis of Htlv-I proviral load in 202 Ham/Tsp patients and 243 asymptomatic Htlv-I carriers: high proviral load strongly predisposes to Ham/Tsp. J. Neurovirol. 4, 586–593. 10.3109/13550289809114225 10065900

[B23] Olindo S. Lézin A. Cabre P. Merle H. Saint-Vil M. Edimonana Kaptue M.. (2005). HTLV-1 proviral load in peripheral blood mononuclear cells quantified in 100 HAM/TSP patients: a marker of disease progression. J. Neurol. Sci. 237, 53–59. 10.1016/j.jns.2005.05.010 15972218

[B24] Orland J. R. Engstrom J. Fridey J. Sacher R. A. Smith J. W. Nass C.. (2003). Prevalence and clinical features of HTLV neurologic disease in the HTLV outcomes study. Neurology 61, 1588–1594. 10.1212/01.WNL.0000096011.92542.DA 14663047

[B25] Otaguiri K. K. dos Santos D. F. Slavov S. N. Depieri L. V. Palma P. V. B. Meirelles F. V.. (2018). TAX -mRNA-carrying exosomes from human T cell lymphotropic virus type 1-infected cells can induce interferon-gamma production *in vitro*. AIDS Res. Hum. Retroviruses 34, 1075–1082. 10.1089/aid.2018.0115 30229663

[B26] Palomino D. C. T. Marti L. C. (2015). Chemokines and immunity. Einstein 13, 469–473. 10.1590/S1679-45082015RB3438 26466066 PMC4943798

[B27] Pinto D. O. Al Sharif S. Mensah G. Cowen M. Khatkar P. Erickson J.. (2021). Extracellular vesicles from HTLV-1 infected cells modulate target cells and viral spread. Retrovirology 18, 6. 10.1186/s12977-021-00550-8 33622348 PMC7901226

[B28] Pinto D. O. DeMarino C. Pleet M. L. Cowen M. Branscome H. Al Sharif S.. (2019). HTLV-1 extracellular vesicles promote cell-to-cell contact. Front. Microbiol. 10, 1–21. 10.3389/fmicb.2019.02147 31620104 PMC6759572

[B29] Poiesz B. J. Ruscetti F. W. Gazdar A. F. Bunn P. A. Minna J. D. Gallo R. C. (1980). Detection and isolation of type C retrovirus particles from fresh and cultured lymphocytes of a patient with cutaneous T-cell lymphoma. Proc. Natl. Acad. Sci. 77, 7415–7419. 10.1073/pnas.77.12.7415 6261256 PMC350514

[B30] Rezaie J. Aslan C. Ahmadi M. Zolbanin N. M. Kashanchi F. Jafari R. (2021). The versatile role of exosomes in human retroviral infections: from immunopathogenesis to clinical application. Cell Biosci. 11, 1–15. 10.1186/s13578-021-00537-0 33451365 PMC7810184

[B31] Rodrigues E. S. Salustiano S. Santos E. V. Slavov S. N. Picanço-Castro V. Maçonetto J. M.. (2022). Monitoring of HTLV-1-associated diseases by proviral load quantification using multiplex real-time PCR. J. Neurovirol. 10.1007/s13365-020-00924-2 35025066

[B32] Saito M. Matsuzaki T. Satou Y. Yasunaga J. Saito K. Arimura K.. (2009). In vivo expression of the HBZ gene of HTLV-1 correlates with proviral load, inflammatory markers and disease severity in HTLV-1 associated myelopathy/tropical spastic paraparesis (HAM/TSP). Retrovirology 6, 19. 10.1186/1742-4690-6-19 19228429 PMC2653460

[B33] Sampey G. C. Saifuddin M. Schwab A. Barclay R. Punya S. Chung M. C.. (2016). Exosomes from HIV-1-infected cells stimulate production of pro-inflammatory cytokines through trans-activating response (TAR) RNA. J. Biol. Chem. 291, 1251–1266. 10.1074/jbc.M115.662171 26553869 PMC4714213

[B34] Théry C. Witwer K. W. Aikawa E. Alcaraz M. J. Anderson J. D. Andriantsitohaina R.. (2018). Minimal information for studies of extracellular vesicles 2018 (MISEV2018): a position statement of the International Society for Extracellular Vesicles and update of the MISEV2014 guidelines. J. Extracell Vesicles 7, 1535750. 10.1080/20013078.2018.1535750 30637094 PMC6322352

[B35] Zhang L. Sharma V. (2000). Human T-cell lymphotropic virus type-I tax gene induces interleukin-8 secretion by autocrine mechanism and has no effect on interleukin-16 in transfected Jurkat cells. Biochem. Biophys. Res. Commun. 273, 865–869. 10.1006/bbrc.2000.3019 10891338

[B36] Zhao T. Yasunaga J. -,i. Satou Y. Nakao M. Takahashi M. Fujii M.. (2009). Human T-cell leukemia virus type 1 bZIP factor selectively suppresses the classical pathway of NF- B. Blood 113, 2755–2764. 10.1182/blood-2008-06-161729 19064727

