# Supplementary material for: The Expression of Tax and HBZ Genes in Serum-Derived Extracellular Vesicles From HTLV-1 Carriers Correlates to Proviral Load and Inflammatory Markers
Source: Front Microbiol. 2022 May 2;13:881634. doi: 10.3389/fmicb.2022.881634 (PMC9108699; doi:10.3389/fmicb.2022.881634)
Supplement: Supplementary file 1 [file Data_Sheet_1.PDF]

Supplementary Material

1 Supplementary Figures

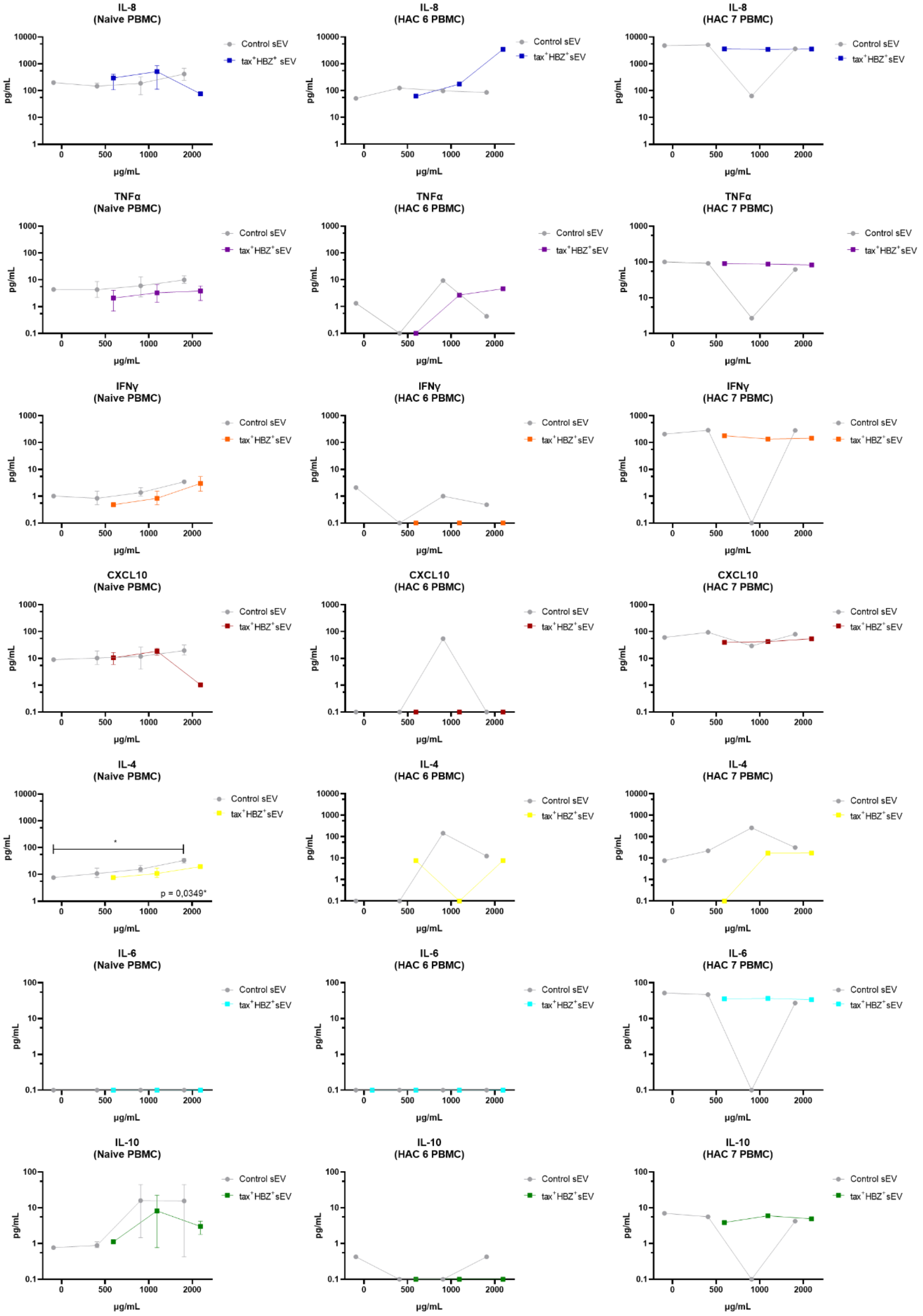

Supplementary Figure 1. Secretion of inflammatory cytokines by naive PBMC and HTLV-1 asymptomatic carriers PBMC (HAC) after exposure to tax+ HBZ+ sEV. Naive PBMC were tested in triplicate. HAC PBMC were tested in unicate.

2 Supplementary Tables

	HAC		HAM/TSP		Control	
**Gender**	**n**	**Frequency (%)**	**n**	**Frequency (%)**	**n**	**Frequency (%)**
**Female**	4	15,4	5	19,2	9	34,6
**Male**	2	7,7	2	7,7	4	15,4
**Total**			26 (100%)			
**Age**						
**< 40**	2	7,7	1	3,8	3	11,5
**≥ 40**	4	15,4	6	23,1	10	38,5
**Total**			26 (100%)			

Supplementary Table 1. Distribution of HTLV-1 carriers according to gender and age.
